# Association between life’s crucial 9 and severe abdominal aortic calcification in U.S. Adults: the mediating role of the systemic inflammatory response index

**DOI:** 10.3389/fendo.2025.1526114

**Published:** 2025-02-28

**Authors:** Kaifeng Tang, Linping Wang, Jinming Ye, Feng Yuan

**Affiliations:** ^1^ Department of Vascular Surgery, Zhejiang Hospital, Hangzhou, China; ^2^ Department of Gynecology, Zhejiang Hospital, Hangzhou, China; ^3^ Department of Thoracic Surgery, Zhejiang Hospital, Hangzhou, China

**Keywords:** abdominal aortic calcification, LC9, NHANES, mediation analysis, systemic inflammatory response index

## Abstract

**Background:**

Life’s Crucial 9 (LC9) is an emerging cardiovascular health scoring system that incorporates Life’s Essential 8 (LE8) alongside mental health factors. However, its relationship with severe abdominal aortic calcification (SAAC) remains poorly understood.

**Objectives:**

The objective of this study is to investigate the relationship between LC9 scores and the incidence of SAAC in the US population.

**Methods:**

Data from 2,323 participants were analyzed, originating from the 2013-2014 National Health and Nutrition Examination Survey (NHANES) cycle. In exploring the dynamics of LC9, its constituents, and their relationship with SAAC, we employed advanced statistical methodologies, specifically multivariable logistic regression and weighted quantile sum regression. Subgroup interaction analyses were conducted to reinforce the conclusions, and mediation analysis was employed to investigate how the systemic inflammatory response index (SIRI) influences the connection between LC9 and SAAC.

**Results:**

In fully adjusted models, an increase of 10 points in LC9 scores was associated with a 26% reduction in the prevalence of SAAC, achieving statistical significance (*P* < 0.001). As LC9 scores increased, a significant decline in SAAC prevalence was noted (*P* < 0.05). The WQS analysis pinpointed strong links between the occurrence of SAAC and variables including exposure to tobacco, blood pressure levels, blood glucose concentrations, and mental health status, the odds ratio stood at 0.244, with the 95% CI extending from 0.119 to 0.495. SIRI was positively correlated with SAAC (*P* < 0.05) and decreased with rising LC9 scores (β = -0.09, *P* < 0.001). Mediation analysis revealed that the SIRI significantly influenced the linkage between LC9 and SAAC, accounting for 5.8% of the mediation effect, with a statistically significant p-value (*P* < 0.001).

**Conclusion:**

This research highlights a robust inverse relationship between elevated LC9 scores and reduced SAAC incidence, suggesting the significant role of LC9 as a key factor in diminishing the frequency of SAAC. Furthermore, SIRI mediates this relationship.

## Introduction

In recent years, health concerns among middle-aged and elderly populations, particularly those related to cardiovascular disease (CVD) mortality, have received growing attention (1). Abdominal aortic calcification (AAC) is commonly recognized as a marker of vascular calcification, which is critically predictive of CVD (2). AAC manifests through the disruption of calcium and phosphorus metabolism and is marked by the irregular accumulation of mineralized plaques within the arterial walls (3). In the United States, the incidence of AAC increases as age advances, affecting 34% of individuals between 45 and 54 years old, and soaring to 94% in those aged 75 to 84 (4). AAC is associated with elevated risks of coronary and cerebrovascular incidents, including a broad spectrum of cardiovascular events and deaths related to CVD (5). Compared to individuals with none or mild AAC, those with SAAC are significantly more susceptible to lethal cardiovascular events, overall cardiovascular incidents, and all-cause mortality (6). However, effective treatment options for AAC remain elusive (7). Identifying risk factors for AAC is, therefore, essential for its prevention.

In 2010, Life’s Simple 7 was introduced by the American Heart Association as a measure to assess cardiovascular health. A decade later (8), in 2020, this evaluation was expanded with the introduction of Life’s Essential 8(LE8), which added sleep health to refine the original metric (9). Previous studies have established the relationship between LE8 and AAC. Cai et al (10) found that higher scores for nicotine exposure, blood glucose, and blood pressure in LE8 were negatively correlated with AAC. Liu et al (11) observed that this relationship appeared to be stronger in patients with a history of chronic kidney disease. Additionally, Ni et al (12)demonstrated that the LE8 score was an accurate tool for assessing cardiovascular mortality. In recent years, the prevalence of depression has significantly increased among patients with cardiovascular diseases (13). Approximately 20% of patients with coronary artery disease (CAD) are affected by depression, while the risk of depression is three times higher in individuals with myocardial infarction or heart failure compared to the general population (14, 15). Meta-analyses have demonstrated that depression or depressive symptoms are significant risk factors for hospitalization or mortality in CAD patients (16). This association remains prominent across various subgroups, including those with acute coronary syndrome, heart failure, stable angina, and those undergoing coronary artery bypass grafting (17). It has been reported that inflammation mediates the relationship between CAD and depression (18). Chronic inflammation promotes AAC, vascular damage, and depression. Inflammatory markers associated with an increased risk of cardiovascular events are significantly elevated in depressed patients with CAD (19, 20). Identifying depression as a standalone risk element for CVD (21), recent studies have introduced a new metric, Life’s Crucial 9 (LC9) (22), which further highlights the importance of mental health in CVD prevention. LC9 includes the following nine components: a healthy diet, regular physical activity, weight management, smoking status (non-smoker or former smoker), blood pressure control, blood glucose levels, cholesterol levels, sleep health, and psychological health (9, 22). Each of these factors is crucial in predicting the overall risk for CVDs and plays a significant role in cardiovascular mortality (23–29). LC9 is a comprehensive measure, incorporating both physiological and lifestyle factors, which has demonstrated a strong predictive value for all-cause cardiovascular mortality (30).

This study provides a more comprehensive perspective on cardiovascular health by investigating LC9, which includes psychological health. Furthermore, it explores the relationship between LC9 and vascular calcification.

## Method

### Study design and population

This study examined data from the 2013-2014 iteration of the National Health and Nutrition Examination Survey (NHANES), a cross-sectional analysis designed to assess the health and nutritional status of the U.S. population. This study collected its data through household interviews and evaluations performed in mobile examination centers. The choice of the 2013-2014 cycle was influenced by the presence of distinctive data on AAC available exclusively during this timeframe.

**Figure 1:**
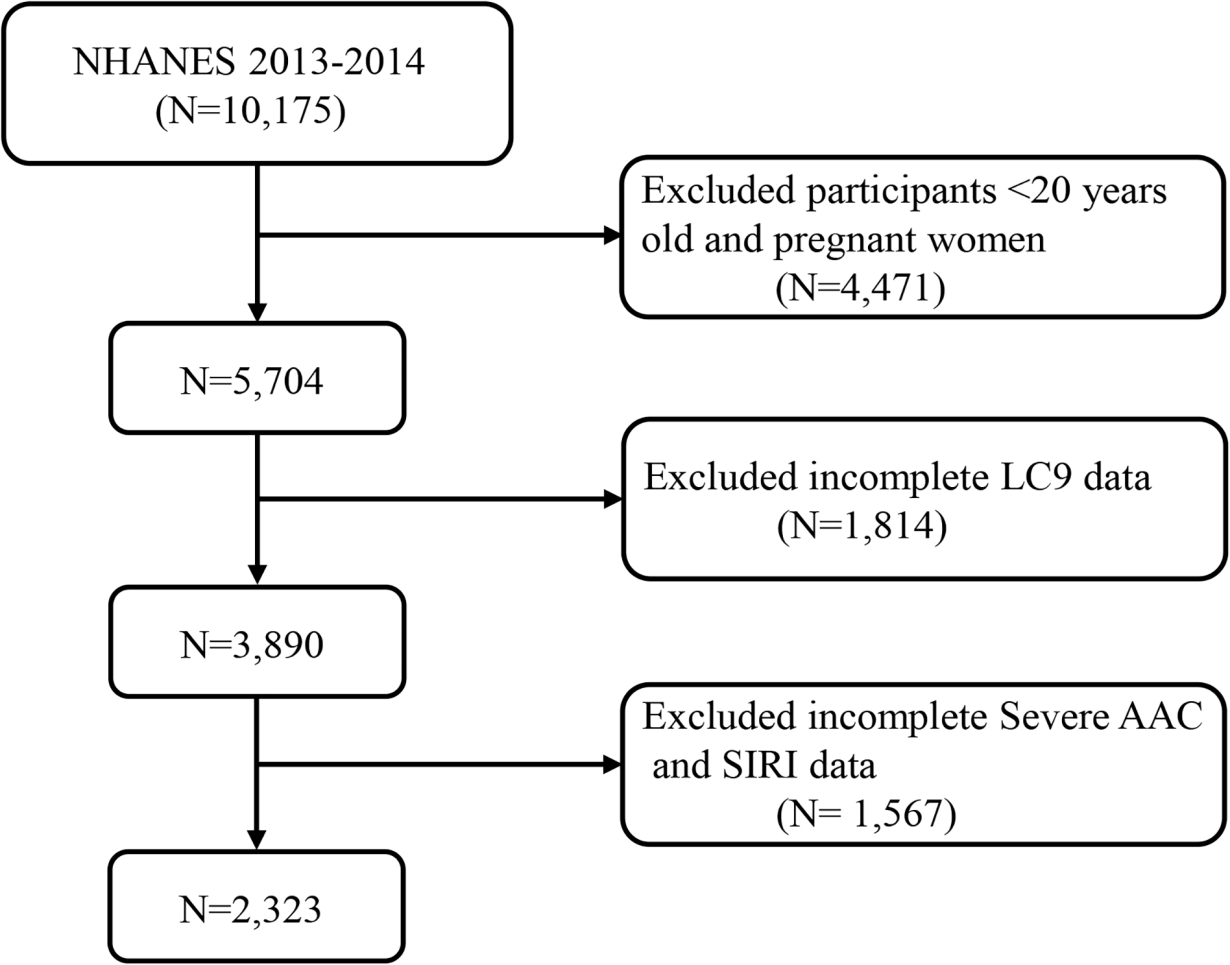
A flow diagram of eligible participant selection in the National Health and Nutrition Examination Survey. AAC, abdominal aortic calcification; CCI, Charlson Comorbidity Index; LC9, Life’s Crucial 9; SIRI, systemic inflammatory response index.

Our study did not include participants younger than 20 years and pregnant women, totaling 4471 exclusions. Additionally, 1814 subjects were omitted due to absent or partial LC9 data, and 1567 were excluded for lacking SAAC or SIRI data. Following these criteria, the study’s final analysis encompassed 2,323 participants. **Figure 1** provides a flow chart detailing the inclusion and exclusion processes for participants.

### Measurement of LC9

LC9 score is derived by adding a depression score to the LE8 score, as detailed in **Supplementary Table S1**. The LE8 score combines eight parameters: four physiological—non-HDL cholesterol, blood glucose, blood pressure, and body mass index—and four behavioral—physical activity, nicotine exposure, sleep duration, and dietary habits. The LE8 algorithm has been described previously (9), utilizing standardized questionnaires for self-reported data on tobacco/nicotine exposure, physical activity, sleep duration, and medication use (31, 32). Dietary intake was assessed using the Healthy Eating Index 2015 (HEI-2015) (33) (**Supplementary Table S2**). Nutritional data were collected from two separate 24-hour dietary recall sessions, and these data were then mapped to USDA food pattern equivalents to calculate the HEI-2015 scores. Physical assessments recorded patient height, weight, and blood pressure. Additionally, evaluations included testing for lipid concentrations and fasting glucose levels, and glycated hemoglobin levels were assessed through blood samples analyzed in a central laboratory.

Depression levels were gauged using the Patient Health Questionnaire-9 (PHQ-9), where elevated scores correspond to more intense symptoms of depression, with scores categorized as follows: 0-4 (100), 5-9 (75), 10-14 (50), 15-19 (25), and 20-27 (0). The LC9 score is calculated by averaging the eight metrics of the LE8 score together with the depression score (34). A higher LC9 score indicates a healthier lifestyle.

### Measurement of SIRI

SII is composed of platelet, neutrophil, and lymphocyte counts, calculated using the formula: platelet counts × neutrophil counts/lymphocyte counts (35, 36). Blood cell counts were measured using an automated hematology analyzer.

### Diagnosis of Sever AAC

AAC was assessed using standard radiologic techniques at UCSF. The semi-quantitative AAC-24 scoring method was used, where higher scores indicated more SAAC. Historical research established three levels of AAC scores: AAC equals zero, a range from mild to moderate calcification (AAC between greater than zero and up to six), and a classification of severe calcification (AAC exceeding six) (37, 38).

Additional information can be accessed at [CDC NHANES 2013-2014] (https://wwwn.cdc.gov/Nchs/Nhanes/2013-2014/DXXAAC_H.htm).

### Covariables

Covariates considered in this study were selected based on previous research (7, 39, 40), the study captured demographic variables such as gender, age, race/ethnicity, education attainment, marital status, and the ratio of family income to poverty (PIR), Charlson Comorbidity Index (CCI) scores, smoking status, alcohol consumption, and obesity. BMI ≥30Kg/m^2^ was defined obesity, smoking status was categorised as never smokers (defined as <100 cigarettes in a lifetime), current smokers (defined as ≥100 cigarettes in a lifetime), and former smoker (defined as ≥100 cigarettes and quit), and Drinking status was defined as heavy drinking (≥4 drinks/day for men, ≥3 drinks/day for women, or ≥5 days of drinking in a month); moderate drinking (≥3 drinks/day for men, ≥2 drinks/day for women, or ≥2 days of drinking in a month); mild drinking (≤2 drinks/day for men, ≤1 drink/day for women, and ≥12 drinks in a year); and never-drinking (total number of drinks in a year <12, and dietary alcohol content of 0%). Definitions and detailed information for each covariate are provided in **Supplementary Tables S3** and **S4**.

### Statistical analyses

Statistical analyses were conducted using R software, version 4.3.1. To ensure national representativeness, NCHS-recommended sample weights were applied. For data weighting, the analysis utilized the weighted two-day dietary sample weight (WTDR2D). To assess categorical variables, weighted chi-square tests calculated p-values, while continuous variables were analyzed using weighted t-tests to determine means and standard deviations (41). Three distinct statistical models were developed: the first, a basic unadjusted model; the second, enhanced by adjustments for demographic factors including age, gender, educational attainment, marital status, race/ethnicity, and PIR; and the third, an extension of the second, which also accounted for behavioral and health variables such as obesity, smoking habits, alcohol use, and the CCI. To investigate the possibility of a linear association between LC9 and SAAC, smooth curve fitting techniques were employed.

For each increment of ten points in LC9, odds ratios (ORs) were computed. Analyses of subgroups, segmented by factors like age, gender, educational attainment, PIR, as well as obesity, smoking, and alcohol consumption habits, were undertaken. Additional refinement of the subgroup analyses was achieved by adjusting for factors such as age, gender, educational level, marital status, PIR and ethnicity.

The R “mediation” package facilitated the assessment of direct, indirect, and total effects. Mediation analysis was performed on 1,000 bootstrap samples with variable correction to assess whether SIRI mediated the relationship between LC9 and SAAC. To determine the mediation proportion, the formula used was:/(indirect effect + direct effect) × 100% (42, 43). The relationship between LC9 and SAAC was dissected through regression analysis, with coefficients illustrating various paths: the aggregate impact (path C), the influence directly traced from LC9 to SAAC, bypassing SIRI (path C’), LC9’s effect on SIRI (path A), SIRI’s subsequent impact on SAAC (path B), and the mediated effect where SIRI channels the influence from LC9 to SAAC (path A*B).

To evaluate the study results from a cardiovascular health perspective, we added detailed data on variables such as tobacco exposure, blood pressure, blood glucose, mental health, physical activity, sleep health, blood lipids, dietary health, and body mass index, assessing their impact on SAAC. Weighted quantile sum (WQS) regression, a method for estimating both the combined and individual contributions of exposures, was utilized to examine the influence of specific factors on LC9. In this approach (44), the dataset was bifurcated randomly, allocating 40% as the training set and the remaining 60% for validation. In the analysis, the training set underwent 1,000 bootstrap replications. Given the inverse relationship of the metric to asthma, the WQS model was executed in reverse order. Metric weights ranged from 0 to 1, summing to 1, and major contributors were identified as indicators with weights greater than 0.1.

Finally, the predictive ability of LS7, LE8, and LC9 for SAAC was compared using receiver operating characteristic (ROC) curves and their respective areas under the curve (AUC). Differences between the AUCs were assessed using the Z-test. A p-value < 0.05 was considered significant.

## Results

### Baseline characteristics of the study population

The research encompassed 2,323 subjects, comprising 53% women and 47% men. Of these, 208 had previously experienced SAAC, whereas 2,115 had no such history. Notably, SAAC was more prevalent in participants 65 years and older, affecting 76% of that group, in contrast to those younger than 65. Additionally, individuals possessing at least a high school diploma exhibited a similarly elevated SAAC prevalence of 82%.

SAAC prevalence exhibited a negative correlation with the family PIR. Factors such as obesity, alcohol consumption, smoking, higher CCI scores, and elevated SIRI scores were all associated with increased SAAC prevalence (*P*< 0.05). Participants exhibiting SAAC presented with reduced LC9 scores, achieving statistical significance (*P*< 0.05). **Table 1** presents a complete overview of baseline characteristics.

**Table 1:** Baseline characteristics of all participants were stratified by Severe AAC.

Characteristic	Overall, N = 2,323 (100%)	Non-Severe AAC, N = 2,115 (91%)	Severe AAC, N = 208 (9%)	P Value
Age (%)				<0.001
* 20-65*	1,607 (71%)	1,557 (75%)	50 (24%)	
*>65*	716 (29%)	558 (25%)	158 (76%)	
Sex (%)				0.44
* Female*	1,226 (53%)	1,113 (53%)	113 (57%)	
* Male*	1,097 (47%)	1,002 (47%)	95 (43%)	
Race (%)				0.089
* Non-Hispanic White*	1,103 (74%)	961 (73%)	142 (81%)	
* Other*	479 (11%)	452 (11%)	27 (10%)	
* Non-Hispanic Black*	445 (9.5%)	421 (9.8%)	24 (5.8%)	
* Mexican American*	296 (6.3%)	281 (6.6%)	15 (3.2%)	
Married/live with partner (%)				<0.001
* no*	812 (31%)	712 (29%)	100 (49%)	
* yes*	1,510 (69%)	1,402 (71%)	108 (51%)	
Education level (%)				0.13
* Below high school*	462 (14%)	418 (13%)	44 (18%)	
* High School or above*	1,860 (86%)	1,696 (87%)	164 (82%)	
PIR (%)				0.18
* Poor*	596 (17%)	537 (17%)	59 (21%)	
* Not Poor*	1,558 (83%)	1,419 (83%)	139 (79%)	
Obesity (%)				0.017
* no*	1,483 (65%)	1,330 (64%)	153 (74%)	
* yes*	840 (35%)	785 (36%)	55 (26%)	
Smoking (%)				<0.001
* never*	1,249 (54%)	1,171 (56%)	78 (36%)	
* former*	670 (30%)	580 (28%)	90 (47%)	
* current*	404 (16%)	364 (16%)	40 (17%)	
Drinking (%)				0.008
* never*	322 (11%)	298 (11%)	24 (11%)	
* former*	484 (18%)	420 (17%)	64 (28%)	
* mild*	879 (41%)	799 (41%)	80 (42%)	
* moderate*	326 (17%)	312 (18%)	14 (6.7%)	
* heavy*	307 (13%)	282 (13%)	25 (12%)	
CCI (mean (SD))	1.33 (1.66)	1.23 (1.60)	2.51 (1.83)	<0.001
SIRI (mean (SD))	1.39 (1.05)	1.37 (1.03)	1.71 (1.16)	<0.001
Mean LC9 score (mean (SD))	70.04 (13.75)	70.50 (13.81)	64.86 (11.88)	<0.001
LC9, Tertile (%)				<0.001
* T1*	865 (33%)	761 (32%)	104 (49%)	
* T2*	825 (34%)	754 (34%)	71 (36%)	
* T3*	633 (33%)	600 (34%)	33 (15%)	
Mean psychological health score (mean (SD))	88.60 (24.39)	88.53 (24.56)	89.40 (22.37)	0.97
Mean HEI-2015 diet score (mean (SD))	45.84 (31.59)	45.90 (31.67)	45.11 (30.73)	0.78
Mean physical activity score (mean (SD))	69.09 (42.67)	70.14 (42.22)	57.37 (45.88)	0.009
Mean tobacco exposure score (mean (SD))	73.94 (36.36)	74.50 (36.37)	67.66 (35.71)	0.007
Mean sleep health score (mean (SD))	83.66 (23.84)	83.42 (24.04)	86.36 (21.30)	0.33
Mean body mass index score (mean (SD))	62.25 (31.12)	61.83 (31.37)	66.90 (27.81)	0.12
Mean blood lipid score (mean (SD))	61.97 (29.58)	62.02 (29.87)	61.41 (26.25)	0.65
Mean blood glucose score (mean (SD))	82.31 (26.05)	83.71 (25.22)	66.69 (29.80)	<0.001
Mean blood pressure score (mean (SD))	62.71 (31.76)	64.49 (31.35)	42.84 (29.51)	<0.001

Mean (SD) for continuous variables: the P value was calculated by the weighted Students T-test.

Percentages (95%CI) for categorical variables: the P value was calculated by the weighted chi-square test.

AAC, abdominal aortic calcification; CCI, Charlson Comorbidity Index; LC9, Life’s Crucial 9; SIRI, systemic inflammatory response index; PIR, poverty income ratio.

### Relationship between LC9 and SAAC

**Figure 2:**
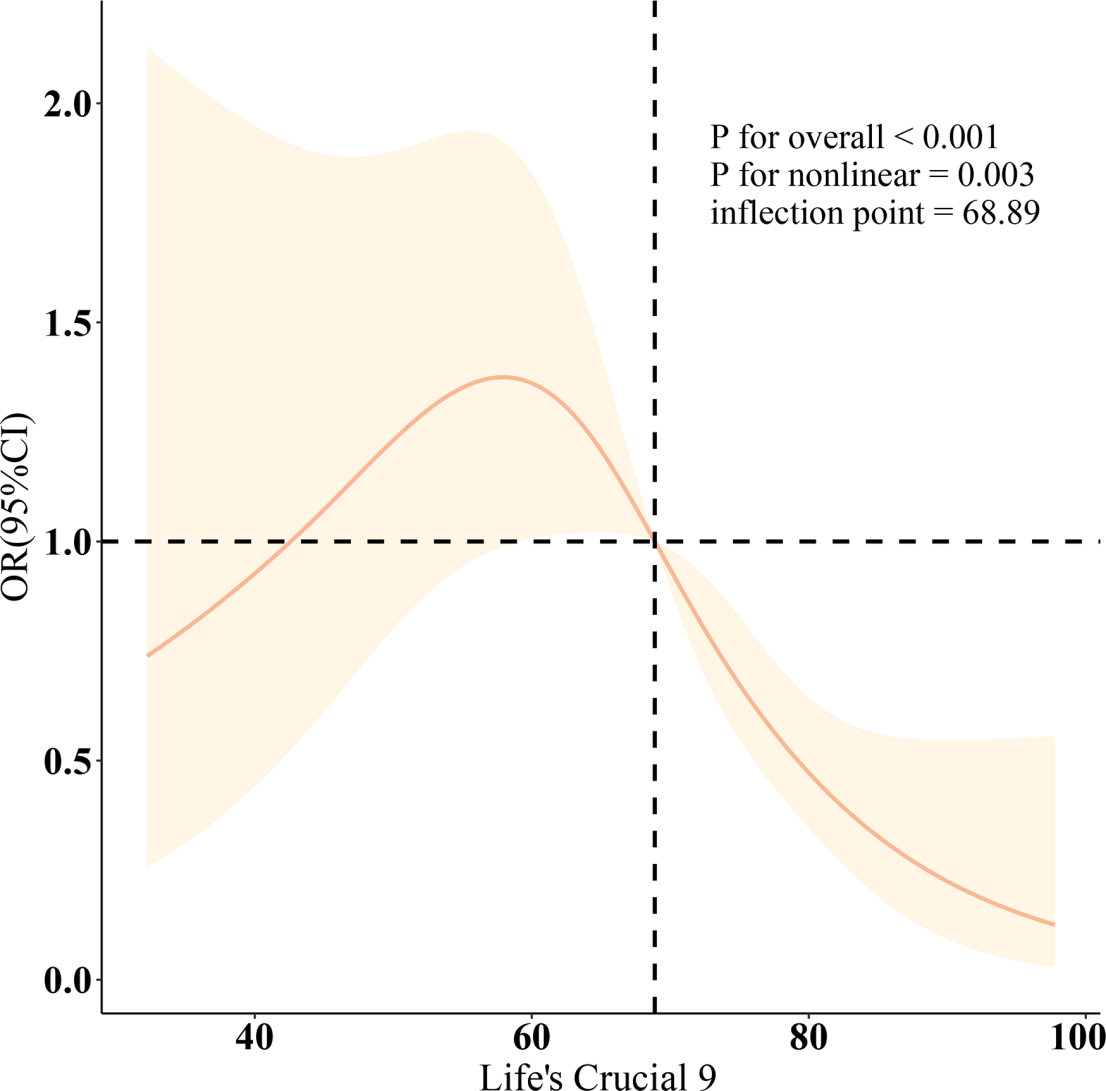
Dose-response relationships between LC9 and Severe AAC. OR (solid lines) and 95% confidence levels (shaded areas) were adjusted for age, sex, education level, marital, PIR, race, obesity, smoking, drinking, and CCI.

**Table 2** displays multivariable logistic regression models that illustrate the association between LC9 and SAAC. Specifically, Model 3 reveals a negative association, with LC9 correlating inversely with SAAC prevalence, evidenced by an OR of 0.74 and a 95% CI ranging from 0.63 to 0.88. The strength of the inverse association between LC9 and SAAC increased across higher tertiles, notably within T2 (OR = 0.59, 95% CI 0.38–0.92) and T3 (OR = 0.29, 95% CI 0.14–0.59), in comparison to the lowest tertile, T1. Statistical tests utilizing T1 as the baseline supported the robustness of these trends (*P*< 0.05). Nonlinear smooth curve fitting illustrated an inverse U-shaped correlation between LC9 and SAAC, identifying a beneficial threshold at 68.89 (*P* for nonlinearity = 0.003), as depicted in **Figure 2**.

**Table 2:** Association between LC9 and Severe AAC.

Characteristics	Model 1 [OR (95% CI)]	*p-value*	Model 2 [OR (95% CI)]	*p-value*	Model 3 [OR (95% CI)]	*p-value*
LC9 - Severe AAC						
Continuous (per 10 scores)	0.75 (0.69,0.82)	<0.001	0.78 (0.67, 0.89)	<0.001	0.74 (0.63, 0.88)	0.001
Tertile						
T1	1 (ref.)		1 (ref.)		1 (ref.)	
T2	0.67 (0.44,1.03)	0.070	0.64 (0.38, 1.08)	0.080	0.59 (0.38, 0.92)	0.020
T3	0.29 (0.18,0.47)	<0.001	0.34 (0.17, 0.69)	0.010	0.29 (0.14, 0.59)	0.002
* P for trend*	<0.001		0.004		0.001	

Model 1: no covariates were adjusted.

Model 2: age, sex, education level, marital, PIR, and race were adjusted.

Model 3: age, sex, education level, marital, PIR, race, obesity, smoking, drinking, and CCI were adjusted.

AAC, abdominal aortic calcification; CCI, Charlson Comorbidity Index; LC9, Life’s Crucial 9; SIRI, systemic inflammatory response index; PIR, poverty income ratio; OR, odds ratio; CI, confidence interval.

### Subgroup and WQS analysis

**Figure 3:**
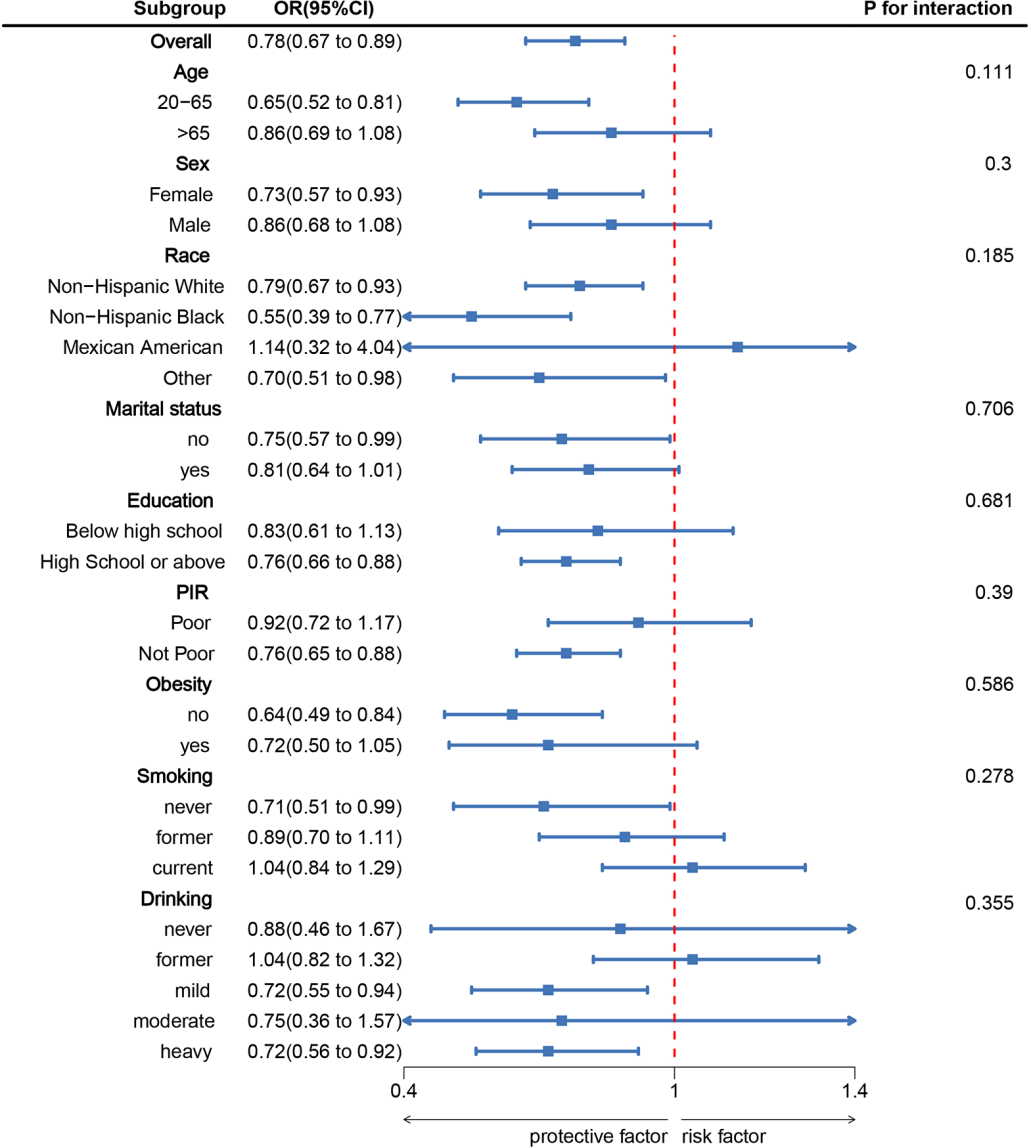
Subgroup analysis between LC9 and Severe AAC. ORs were calculated per 10-unit increase in LC9. Analyses were adjusted for age, sex, education level, marital, PIR, and race.

**Figure 3** presents subgroup analyses depicting the link between LC9 and SAAC, factoring in demographic and behavioral variables such as age, sex, educational attainment, marital status, race, income, and lifestyle factors like obesity, smoking, and alcohol use. These analyses evaluate the interaction effects and the consistency of the relationship. Investigative results consistently showed a persistent inverse relationship between LC9 and SAAC across various subgroups, with the absence of notable interactions concerning stratified variables (*P* > 0.05). This stability underscores the robustness of the association across various demographics.

**Figure 4:**
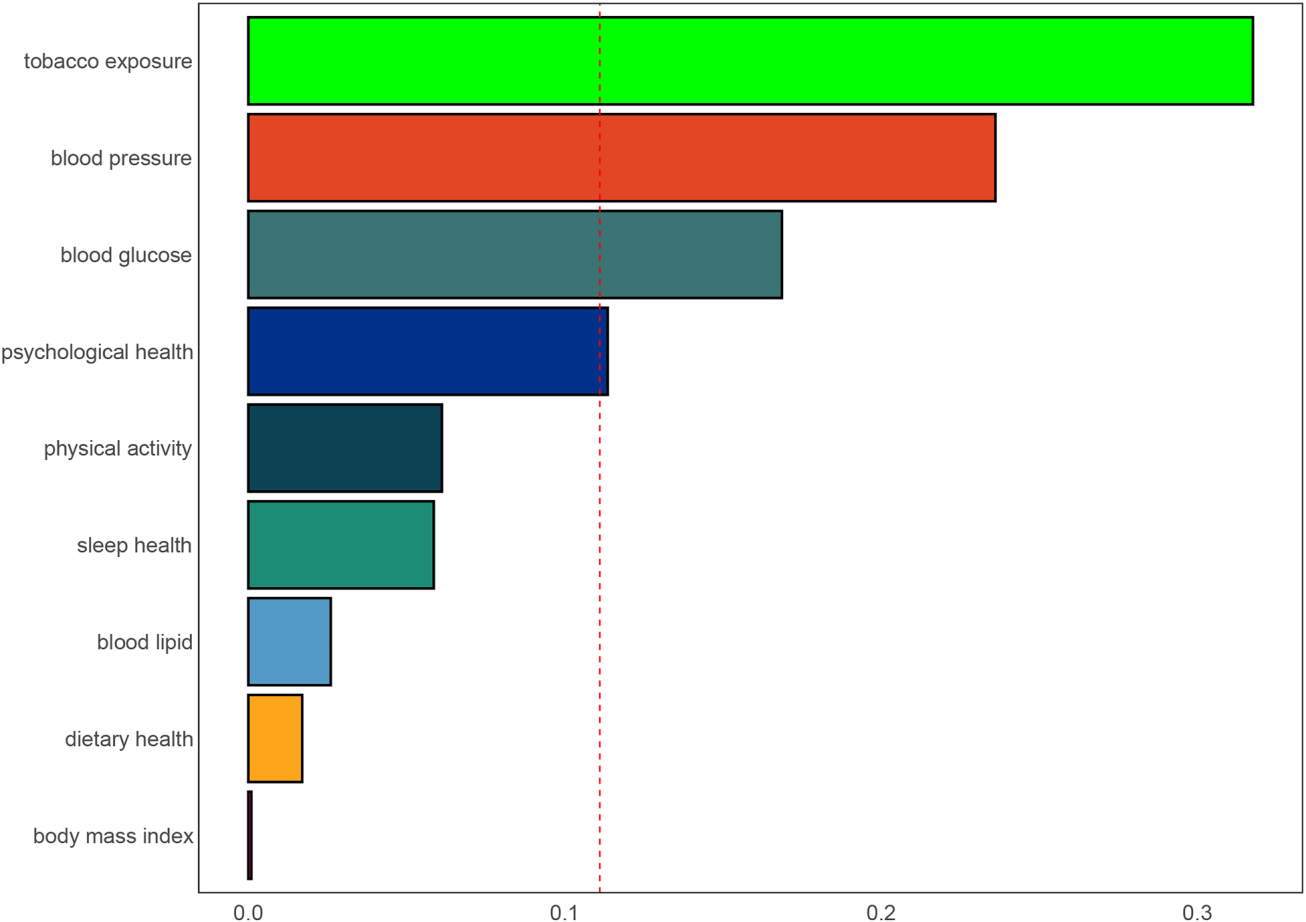
Weights represent the proportion of partial effect for each LC9 metric in the WQS regression. Model adjusted for age, sex, education level, marital, PIR, and race.

The applied regression model, the WQS, revealed a robust negative association between the WQS index and the likelihood of SAAC. This relationship is substantiated by an OR of 0.244, with a 95% CI extending from 0.119 to 0.495, as specified in **Supplementary Table S5**. Analysis depicted in **Figure 4** highlighted tobacco exposure as the primary contributor to SAAC risk, carrying a weight of 0.318. This was followed by blood pressure, blood glucose and psychological health, which had respective weights of 0.236, 0.169 and 0.114, suggesting their lesser yet notable impact on SAAC likelihood.

### Mediation effect

**Table 3** presents the relationships between LC9, SIRI, and SAAC, which form the basis for the mediation analysis. When treated as a continuous variable, SIRI was positively associated with SAAC (OR = 1.25, 95% CI: 1.08–1.44, *P* < 0.05). Controlling for various covariates, the analysis identified a clear inverse relationship between LC9 and SIRI, demonstrated by a regression coefficient (β) of -0.09 and a 95% CI ranging from -0.13 to -0.05, achieving statistical significance with a p-value less than 0.001. This relationship qualifies for further exploration through mediation analysis.

**Table 3:** Association between LC9, SIRI, and Severe AAC.

	β/OR	95%CI	P-value
LC9 - SIRI	-0.09	(-0.13, -0.05)	<0.001
SIRI - Severe AAC	1.25	(1.08, 1.44)	0.005

AAC, abdominal aortic calcification; LC9, Life’s Crucial 9; SIRI, systemic inflammatory response index.

LC9, SAAC, and SIRI were used as the independent variable, dependent variable, and mediator, respectively, in the mediation analysis. Mediation analysis reveals that SIRI explains 5.8% of the association between LC9 and SAAC. The indirect effect was quantified at -3.84×10^-3^(95%CI: -7.20×10^-3^, -1.01×10^-3^), with a significance level of *P* = 0.004. Additionally, the direct effect of LC9 on SAAC was recorded at -6.16 × 10^-2^(95%CI: -8.75×10^-2^, -3.70×10^-2^)with a p-value < 0.001, the total effect of LC9 on SAAC was -6.54×10^-2^(-9.15×10^-2^, -4.08×10^-2^)with a p-value <0.001. Calculating the mediation proportion involved dividing the indirect effect by the sum of both indirect and direct effects, and then multiplying by 100% (*P* = 0.004). This computation supports the role of SIRI as a mediator in the linkage between LC9 and SAAC, as detailed in **Supplementary Figure S1**, **Supplementary Table S6**.

### Comparison of LS7, LE8 and LC9 in predicting SAAC

The ROC curve results for the three metrics are shown in **Supplementary Figure S2**. The AUC for LC9 in predicting SAAC was 0.648 (95% CI: 0.612-0.683), which was significantly superior to LS7 and LE8. The difference was statistically significant (*P* < 0.001).

## Discussion

Analysis of data from the NHANES database revealed that LC9 had a U-shaped inverse correlation with the prevalence of SAAC when adjustments were made for pertinent covariates. Moreover, the SIRI appears to mediate the observed relationship between LC9 and SAAC. The WQS analysis highlights that the emergence of SAAC is significantly influenced by several factors including exposure to tobacco, blood pressure levels, blood glucose concentrations, and psychological health conditions. It is worth noting that the first three factors are components of LE8, which further confirms the involvement of LE8 in the relationship between LC9 and SAAC. Additionally, the psychological health component, which is not included in LE8, also contributes to this relationship. We believe that LC9 provides a more comprehensive perspective in assessing cardiovascular health, potentially revealing a closer association with SAAC.

Research findings consistently reveal a correlation between AAC and the LS7 and LE8 scores, corroborating previous studies that have identified these associations (10, 11, 25, 45), LC9 builds upon LE8 by incorporating the influence of depression. The relationship between smoking and arterial calcification has been well established, and LE8 accounts for both active and passive smoking (46, 47). Elevated blood glucose levels in diabetic conditions are linked to the calcification of vascular smooth muscle cells, which contributes to vascular calcification (48, 49). Elevated blood pressure, a well-known predictor of CVD, is closely linked to arterial systolic pressure and vascular calcification (50). Recent research has shown that LC9, which includes a depression score, outperforms LE8 in predicting cardiovascular and all-cause mortality (30). Meta-analysis studies confirm that depression independently increases the likelihood of morbidity and mortality related to CVDs (16, 51–53), showing a 30% increase in the incidence of myocardial infarction and coronary heart disease (52). Harshfield et al. conducted a comprehensive analysis involving 563,255 individuals across 22 cohorts, revealing that even subclinical levels of depressive symptoms at baseline are linked to the onset of CVD (54). Analysis of NHANES data supports the link between symptoms of depression and elevated cardiovascular mortality risk (55–57). Studies suggest the link is particularly strong among women, individuals with high dietary cholesterol, lower income households, or those diagnosed with diabetes (58, 59). Potential mechanisms linking depression and CVD include endothelial dysfunction, alterations in the autonomic nervous system, neurohormonal changes, clotting factors, platelet function, and proinflammatory cytokines (13, 60, 61). The underlying physiological factors for sex-based disparities in this context likely include the impact of sex hormones on the regulation of the hypothalamic-pituitary-adrenal (HPA) axis (62). AAC is indicative of subclinical atherosclerosis and forecasts future cardiovascular issues, underscoring the urgency for intensified primary prevention in at-risk populations. In recent years, there has been increasing attention on the relationship between mental health and cardiovascular diseases. Certain personality traits, such as high levels of anger, hostility, anxiety, and depression, have been associated with elevated C-reactive protein(CRP) levels and sympathetic nervous activation, both of which contribute to an increased risk of atherosclerosis (63, 64). A cross-sectional study based on a Swedish population demonstrated that higher scores on the Life Orientation Test-Revised (LOT-R) were associated with lower coronary artery scores (65). In fact, individuals with better mental health are often socially advantaged, with higher socioeconomic status and healthier lifestyles, such as regular exercise, balanced diets, and avoidance of smoking (66). Some studies suggest that if risk factors for vascular stiffening, such as dietary habits and physical activity patterns, are difficult to modify, improving mental health may help promote healthier behaviors (67). Such healthier behaviors are often associated with a reduced risk of cardiovascular diseases, such as atherosclerosis.

The research indicates that SIRI could be the intermediary in the relationship between LC9 and SAAC, likely via pathways associated with nicotine exposure as accounted for in the LC9 scoring framework. SIRI as a novel inflammatory index, has been shown to be significantly associated with cardiovascular mortality and morbidity. Xia et al (68) found that elevated SIRI levels were associated with an increased risk of cardiovascular death, a finding that has also been confirmed in patients with obesity, osteoarthritis, rheumatoid arthritis, and hypertension (69–71). Gu et al. (72) demonstrated that this relationship is even more pronounced in patients with chronic kidney disease, with the risk increasing by as much as 150%. Studies have shown that smokers have elevated levels of neutrophils, lymphocytes, and monocytes compared to non-smokers (73), exposure to both direct smoking and secondhand smoke is linked to elevated levels of serum CRP (74). Moreover, tobacco smoke activates stress-activated kinases and mitogens in human fibroblasts and pulmonary epithelial cells, Activation of NF-κB-dependent inflammatory genes such as IL-6, IL-8, and COX-2 occurs alongside alterations in chromatin structure (75). Cigarette smoke triggers the activation of the NLRP3 inflammasome in atherosclerosis, engaging monocytes, macrophages, and foam cells in the process (76). In studies involving rats, the introduction of nicotine into their systems results in elevated CRP concentrations specifically within the vascular smooth muscle cells and aortic tissues. This is accompanied by a marked increase in reactive oxygen species (ROS) and triggers the activation of the NLRP3 inflammasome (77). Nicotine induces the activation of the NLRP3 inflammasome through ROS pathways, resulting in pyroptosis and the secretion of IL-1β and IL-18 in human aortic endothelial cells (78).

In a comprehensive analysis using multivariable logistic regression, it was determined that LC9 is inversely associated with SAAC. Further, through the application of smooth curve fitting techniques, data illustrated a U-shaped correlation. Notably, the point of maximum benefit was identified at a threshold value of 68.89. Upon controlling for factors such as age, sex, race, marital status, education, income, obesity, smoking, and drinking, subgroup analysis showed no statistically significant interactions between LC9 and these variables (*P* > 0.05), indicating a consistent relationship across different strata.

The findings of this study highlight the strong relationship between LC9 and the prevalence of SAAC in U.S. adults. Our results suggest that higher LC9 scores, which reflect a combination of cardiovascular health metrics and mental health factors, are associated with a significantly lower prevalence of SAAC. These findings offer several practical applications and implications for clinical practice and public health. In clinical practice, healthcare providers can use LC9 scores as a simple yet effective tool for identifying individuals at higher risk of developing SAAC and potentially preventing associated cardiovascular events. By focusing on improving lifestyle factors such as diet, physical activity, and mental health, as well as controlling blood pressure and blood glucose levels, interventions can be tailored to reduce the burden of vascular calcification. These efforts may, in turn, lead to reduced cardiovascular morbidity and mortality. For example, early identification of individuals with lower LC9 scores could prompt interventions aimed at improving both physical and mental health, which could have long-term benefits in preventing SAAC and CVD. Our study expands upon these findings by incorporating mental health factors in the risk assessment of SAAC. This adds a critical layer to cardiovascular health evaluation, potentially leading to more comprehensive strategies for prevention and treatment. The inclusion of SIRI as a mediator also provides new insights into how inflammation may contribute to the development of SAAC, suggesting potential new therapeutic intervention pathways.

Several limitations are present in this research. Firstly, the design—cross-sectional in nature—precludes the determination of causal relationships. While it is hypothesized that LC9 may affect SAAC through the SIRI pathway, further longitudinal research is required to verify this link. Additionally, self-reported health behaviors are subject to bias, which requires careful interpretation of the results. Although NHanes employed a stratified probability sampling method using a complex, multistage stratified probability sampling approach that is theoretically representative of the US deinstitutionalised population, this may limit the generalisability of this study’s findings to many countries around the globe. Finally although we controlled for several potential confounders, we may still be subject to potential confounding effects and issues related to multiple dependent variables. As different components of the LC9 such as (physical activity, sleep and diet) were measured at different levels, the interrelationships between the variables may not have been fully considered, and future studies need to explore potentially possible interactions between the variables using more refined statistical methods.

The research identifies a correlation between LC9 scores and the incidence of SAAC, suggesting the utility of LC9 in predicting SAAC risk.

## Conclusion

The findings of our investigation reveal a notable link between LC9 and SAAC, with SIRI serving as an intermediary. Elevated LC9 levels potentially decrease SAAC occurrences. To delve deeper into the mechanisms at play, additional longitudinal research and basic experiments are essential.

## Data Availability

The raw data supporting the conclusions of this article will be made available by the authors, without undue reservation.
